# Case of Premature Puberty

**Published:** 1811-02

**Authors:** A. Cookson

**Affiliations:** Lincoln


### *To the Editors of the Medical and Physical Journal.*

### *Queries on Cancer.*

GENTLEMEN,

### *Case of Premature Puberty.*

GENTLEMEN,

A FEW days after I had perused the very singular instances of premature puberty detailed in a former Number, the following most extraordinary instance occurred, of which I have had ocular demonstration.

Charlotte Mavor, of this city, born in the month of March, 1806, had an appearance of the catamenia at the age of three years and a half, which recurred at uncertain and unequal periods till within six months ; since which period she has had them regularly every four or five weeks. Of late they have flowed in considerable quantity, being of a rich a rich florid colour. She experiences no pains or other symptoms indicating their approach, save a little stretching and yawning. She is a strong-built womanly kind of child, her nates and shoulders being as large and as broad as those of a grown-up woman. The mammæ are uncommonly large and protuberant, and the pudenda as prominent as those of a girl of sixteen; the mons veneris and labia being furnished with a downy kind of hair. Until within these few weeks the child has enjoyed a good state of health, but since that time she seems to have drooped and languished, owing, no doubt, to the great periodical loss she experiences, and which her young and tender frame is unequal to sustain. I do not find that this girl has exhibited any particular marks of attachment to the other sex; but have thought it right to caution the mother on this head; though I am apprehensive she will not survive many years.

It may be a matter of curious speculation, whether this child could be impregnated, conceive, and produce her kind—I am inclined to think in the affirmative.

I remain, Gentlemen, &c.

A. COOKSON, M. D.

*Lincoln, December* 10, 1810.

